# Regulation of microRNAs function by circular RNAs in human cancer

**DOI:** 10.18632/oncotarget.19930

**Published:** 2017-08-04

**Authors:** Chao Han, Nicole A. Seebacher, Francis J. Hornicek, Quancheng Kan, Zhenfeng Duan

**Affiliations:** ^1^ Department of Pharmacy, The First Affiliated Hospital of Zhengzhou University, Zhengzhou, P.R. China; ^2^ Sarcoma Biology Laboratory, Center for Sarcoma and Connective Tissue Oncology, Massachusetts General Hospital and Harvard Medical School, Boston, MA, USA

**Keywords:** circRNA, miRNA, cancer, gene regulatory mechanisms, target therapy

## Abstract

Circular RNAs (circRNAs) are a newly validated class of endogenous non-coding RNA, generated from the ligation of exons, introns, or both, which arise via a diverse number of cellular mechanisms. Due to rapid advances in the development of combined high-throughput sequencing and bioinformatics analyzing tools, many circRNAs have recently been discovered, revealing an expansive number of ubiquitously expressed mammalian circRNAs. Interestingly, it has recently been confirmed that circRNAs bind to microRNAs (miRs), as miR “sponges”, acting to suppress miR function. As miRs are known to alter the development and progression of cancer, circRNAs may offer a novel diagnostic and prognostic biomarker for cancer. Indeed, recent evidence has shown that circRNAs are associated with many human cancers. Herein, we review the molecular characteristics and biogenesis of circRNAs, with a focus on newly identified circRNAs that may play an important role in human cancer, through their regulation of miR expression.

## INTRODUCTION

Circular RNAs (circRNAs) have recently been identified as naturally occurring alternative RNA splicing products, which can inhibit the expression of microRNAs (miRs) in eukaryotic cells [1–4]. These miRs are small non-coding RNAs that bind to the 3′-untranslated regions (3′-UTR) of target messenger RNAs (mRNAs), which functions to repress their translation, thereby potentially regulating an extensive set of biological processes [5]. Emerging evidence has demonstrated that circRNAs are closely associated with a number of human diseases, most notably, cancer [6, 7]. The mechanisms of miRs expression control can occur at the epigenetic, transcriptional, and post-transcriptional level [8–10]. Inhibition of miR activity by antisense oligonucleotides or antagomirs has been used to study the functions of miRs, both *in vitro* and *in vivo* [11, 12]. Recently, it was found that circRNAs can serve as competitive inhibitors by binding to miRs, referred to as “miR sponges”, or they may act as target mimics to block the activity of a specific miR [2, 4, 13, 14]. The circRNAs contain binding sites for the miR either in the non-coding transcript or in the 3′- UTR of a specific gene [15]. High throughput RNA sequencing and bioinformatic data analysis have predicted thousands of circRNAs in the human genome, suggesting that they may function as important post-transcriptional regulators [4, 16, 17]. Indeed, the down-regulation of miR expression by circRNAs, particularly their role as miR sponges, has recently been investigated in numerous different human cancers, such as lung, gastric, and colorectal cancers [18, 19]. Collectively, this data has brought to light a novel suppressive role for circRNAs in miR expression and, consequently, altered activity of their downstream target genes [20, 21]. Herein, we review the characterization, biogenesis and classification of circRNA, with a special focus on recent studies which have explored the regulation of miR expression by circRNA in human cancer.

## IDENTIFICATION AND CHARACTERIZATION OF circRNAS

CircRNAs were initially described as a naturally occurring family of non-coding RNAs, ubiquitously expressed in mammalian cells [22]. Soon after, researchers began to demonstrate that some of these circRNAs could in fact be translated [23]. One recent example of this is circ-ZNF609, which contains an open reading frame spanning from the start codon and ending at an in-frame STOP codon, similar to the linear transcript, but formed by circularization. It is translated into a protein in a cap-independent and splicing-dependent manner, becoming an example of a protein-coding circRNA in eukaryotes, which functions in myogenesis [24]. Recently, it has been shown that the expression of circRNAs is widespread, with circRNA expression accounting for greater than 10% of gene expression [25–27]. Compared with linear RNA, such as mRNA or long non-coding RNA (lncRNA), circRNAs are circular in nature. This single-stranded covalently-closed circular structure was first revealed in 1976 in plant viroids, which are small pathogens known to exert similar biological functions to viruses [28]. This was closely followed in 1979 by the identification of circRNAs in the cytoplasm of eukaryotic cells, and in 1991 the first report human circRNA was published [29, 30]. These species had generally been considered to be of very low abundance and were likely representing errors in splicing [25]. It was over a decade later before it was revealed that a large number of circRNAs may actually exist. So far, there is no evidence to suggest that circRNAs are translated, even though most overlap protein-coding sequences [26].

The synthesis of circRNAs is mostly thought to occur by forming a covalent 3′,5′-phosphodiester bond from alternative back-splicing of exons and/or introns in precursor messenger RNA (pre-mRNA) [22, 31, 32]. As a consequence of this, the most prominent difference between circRNAs and linear RNAs is that circRNAs do not have a 5′ cap and a 3′ polyadenylated tail structure [22]. Based on their specific structure, circRNAs are much more stable than their linear counterparts; consequently, they are not degraded easily by Ribonuclease R (RNase R) or RNA exonuclease activity [25, 33]. Indeed, most circRNA species exhibit a half-life of over 48 hours, compared to an average half-life of 10 hours for mRNAs [22].

A recent study of human circRNAs has revealed that these molecules are usually composed of 1–5 exons and unlike miRs or lncRNAs that are approximately 22 and 200 nucleotides (nt) in length, respectively [5, 13]. Human circRNAs generally vary in length from less than 100 nt to over 4000 nt [34]. As a consequence of this diversity in length and structure, circRNAs are likely to have an expansive and complex range of mechanisms and biological functions, most of which are yet to be identified. Moreover, circRNAs have also shown variability in cellular localization, which may be linked to their functional mechanisms. Notably, circRNAs which have sponge activity have been consistently localized to the cytoplasm [25, 35].

In addition to this, cross-species comparisons using genome-wide analyses have shown that sites of circularization are conserved across species at a rate above that expected by chance [3, 35]. Furthermore, circRNAs also demonstrate tissue and developmental stage-specific expression [36]. This, in turn, suggests that circRNAs may have specific physiological roles within cells. Several possible functions have been proposed, including miR binding, miR transport, protein binding, regulation of protein transcription and translation [2, 25, 37].

## THE BIOGENESIS AND CLASSIFICATION OF circRNAS

**Figure 1:**
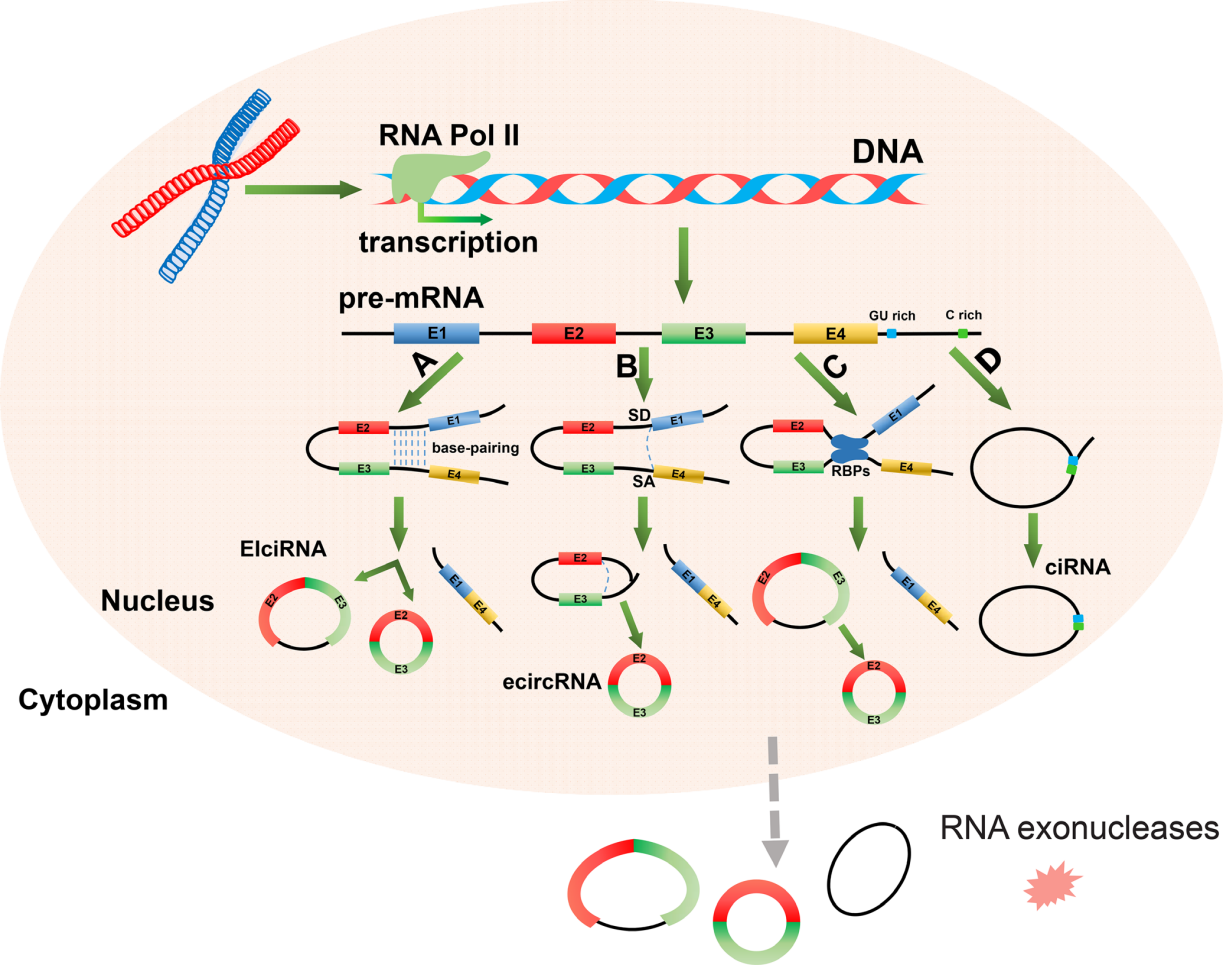
The four potential mechanisms of circRNAs biogenesis In the nucleus of a eukaryocytic cell, precursor messenger RNA (pre-mRNA) is firstly transcribed from DNA by RNA polymerase II (RNA Pol II). Following this, circRNAs are formed through the non-canonical splicing of this pre-mRNA. (**A**) Intron-pairing-driven circularization. Two connected introns form a circular structure *via* base-pairing. Introns are then either eliminated or retained to form exon circular RNA (ecircRNA) or exon-intron circular RNA (EIciRNA). (**B**) Lariat-driven circularization. Exon skipping occurs when a splice donor (SD) site in the 3′ end of exon 1 covalently links to a splice acceptor (SA) site in the 5′ end of exon 4. This forms a lariat structure containing exon 2 and exon 3. CircRNAs and linear RNAs are formed once introns are removed. (**C**) RBP-driven circularization. Binding of RBPs (blue box) creates a bridge between two flanking introns. The circRNA is then formed after introns are removed. (**D**) Circular intron RNA (ciNRA). The intron-derived circRNAs form when intron lariats escape the usual intron debranching and degeneration processes, and thus form stable circular intronic RNAs (ciRNAs) Sequences near the 5-splice site (blue box), which is rich in “GU” sequences, and the branch point (green box), which is rich in “C” residues, allow for an intron to escape debranching, generating the ciRNA. CircRNAs are generally located in the cytoplasm and are relatively stable owing to their resistance to RNA exonucleases.

Most circRNAs are produced from host genes and contain complete exons [23]. The biogenesis of circRNAs involves pre-mRNA transcription by RNA polymerase II (RNA pol II), and back-splicing mediated by spliceosomal machinery [27, 38]. Recent studies have indicated that the biogenesis of circRNAs, through the back-splicing of pre-mRNA, requires different mechanisms than those used for the canonical splicing of linear RNAs [27, 39]. There are four proposed circularization models for circRNAs synthesis (Figure 1) [39, 40]. The first model of circRNAs synthesis is intron-pairing-driven circularization, termed “direct back-splicing” (Figure 1A). It has been suggested that intronic motifs might border circularized exons and upon joining, *via* base pairing, they form the fused circRNA. It has been proposed that these bordering intronic sequences are generally very long (∼15,000 nt) and are almost perfectly complementary, which is commonly referred to as reverse complementary matches (RCMs). Consequently, this promotes the hairpin formation of the transcript [41]. This helps to explain how the two tails of the pre-mRNA can be in spatial proximity, prior to splicing. Notably, it has been reported that in humans the bordering introns of circRNAs are highly enriched in ALU repeats, which contain RCMs [25]. More recent studies have further verified the model of intron-pairing-driven circularization [17, 33, 41, 42]. The second model of circRNA synthesis is termed the lariat-driven circularization or exon skipping model (Figure 1B) [39]. In an exon skipping event, a 3′ splice donor (SD) site of an upstream exon covalently joins to the 5′ splice acceptor (SA) of a downstream exon, thereby forming a lariat structure. This lariat may then be internally spliced, and the intronic sequences deleted or preserved. This leads to the generation of either exon circRNAs (ecircRNAs) or exon-intron circRNAs (EIciRNAs) [43, 44]. The third model of circRNAs synthesis is termed the RNA binding protein (RBP) driven circularization model (Figure 1C). In this model, the alternative splicing factors, Quaking (QKI) and Muscleblind (MBL), bind certain circRNA flanking introns and act as RBPs to bring two flanking intronic sequences close together, inducing circularization of genes that contain binding sites for RBPs in their introns [45]. This mechanism is similar to the intron-pairing-driven circularization pathway, except that RBPs regulate adjacent splice sites instead of the direct base pairing between complementary motifs seen in the intron-pairing-driven model. In addition to this, binding of RBPs may also promote circularization by inhibiting canonical splicing or by stabilizing complementary sequences [38, 45]. The fourth model of circRNAs synthesis is spliceosome-mediated machinery driven circularization (Figure 1D). In this model, stable circular intronic RNAs (ciRNAs) result when intron lariats escape the normal intron debranching and degeneration [46]. The process depends on the presence of a 7 nt GU-rich motif near the 5′ splice site and a 11 nt C-rich motifs near the branchpoint site [46, 47].

## FUNCTION OF circRNAS AS SPONGES OF miRs IN HUMAN CANCER

**Figure 2:**
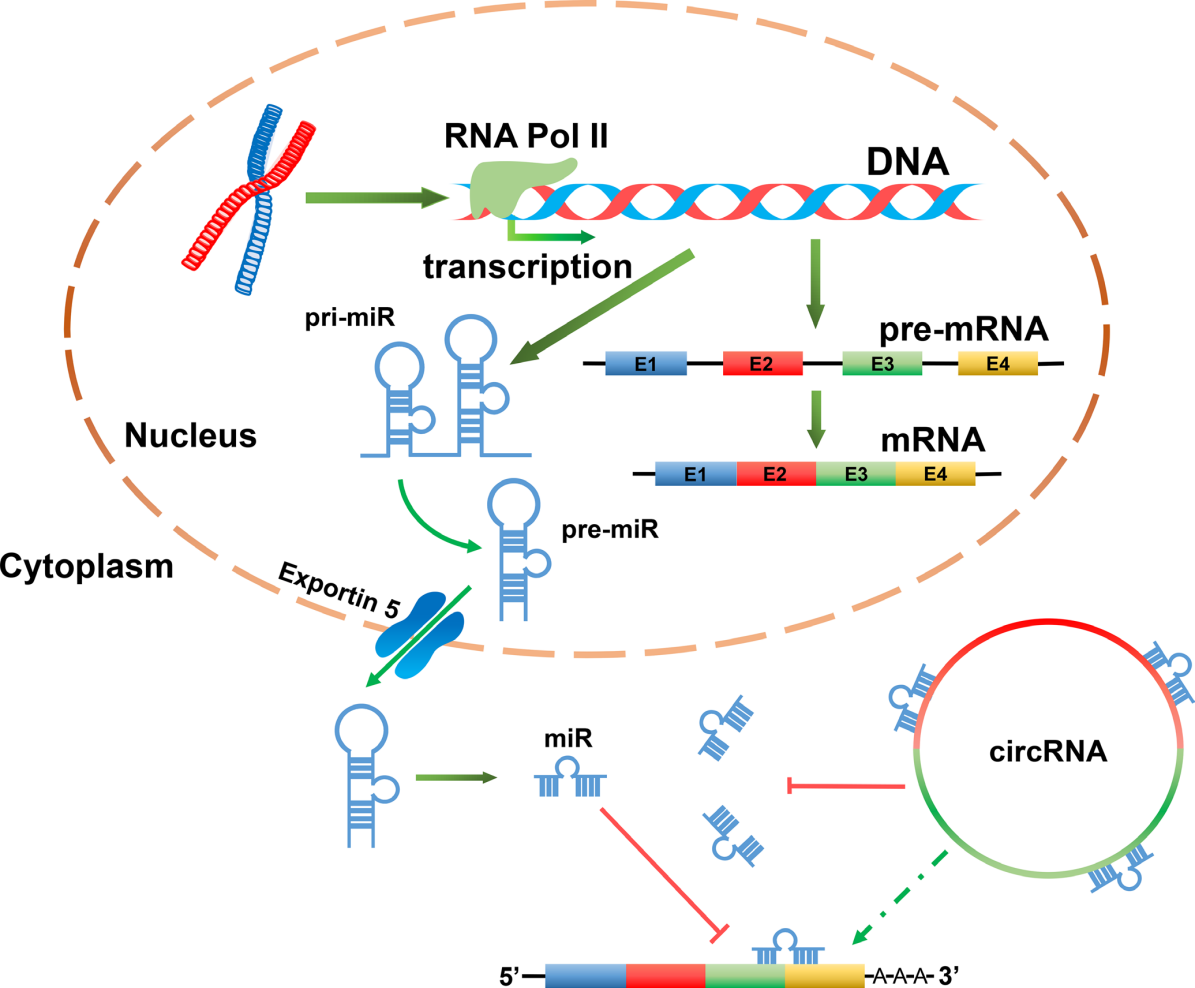
CircRNAs acting as miR sponges can indirectly regulate target genes of miR In the nucleus, miR genes are transcribed by RNA pol II to generate primary miR (pri-miR). These pri-miRs are then processed into precursor miRs (pre-miRs) and transported into the cytoplasm where they form mature miRs. A mature miR can bind to the 3′-UTR of a mRNA, thereby suppressing its translation or result in its cleavage. A circRNA may be rich in miR response elements (MREs). As a result of this, circRNA and mRNA can compete for the same miR. Therefore, it is likely that circRNAs can indirectly regulate the expression of mRNAs which containing the same miR binding sites.

One of the most important functions ascribed to circRNAs is their ability to bind to miRs as a competing endogenous RNAs (ceRNAs), thereby repressing the function of the miRs (Figure 2) [15, 25, 39]. Indeed, circRNAs have been shown to have multiple miR response elements (MREs) [2]. For instance, in one study of CDR1as/ciRS-7, a circRNA sponge for miR-7 over 60 conserved seed match segments were identified, suggesting very dense binding [2, 48]. Due to the growing interest in this field, the cellular functions of sponge miRs are currently being investigated [20, 21]. Interestingly, reports have suggested that depending on the cellular localization of circRNAs, they may have different functional roles. For example, in the nucleus, circRNAs can form large RNA-protein complexes (RPCs) through their binding to RNA binding proteins (RBPs), which will influence the transcription of local linear RNA [49–51]. CircRNAs can also function in gene regulation by competing with linear pre-mRNA splicing. For instance, circ*Mbl* has the capacity to sponge muscleblind (MBL), by regulating the splicing of its own pre-mRNA into a circ*Mbl* or translatable mRNA [38]. While the multitude of potential functions exhibited by circRNA remains to be fully elucidated, circRNAs are known to participate in a plethora of biological processes. To date, all known circRNAs displaying sponge activity have been localized to the cytoplasm, which may be an important feature in their functional role (Figure 2) [15].

CircRNA also have been shown to be associated with several diseases, most notably cancer [20, 52–54]. At present, it is known that there exists a specific association pattern between circRNAs and miRs in cancer [18, 19]. Current evidence suggests that circRNAs may enhance or suppress the progression of cancer through suppressing miR species that are associated with proliferation, differentiation, migration and carcinogenesis [27].

### CircRNAs in lung cancer

The circRNA, circRNA-ITCH (*cir-ITCH*), has been reported to play an important role in inhibiting lung cancer progression, by functioning as an miR sponge of mulitple oncogenic miRs. This acts to enhance the expression of E3 ubiquitin-protein ligase (ITCH) and thus suppress activation of Wnt/β-catenin signaling, which is an important pathway involved in the process of cellular proliferation and migration. *Cir-ITCH* has been verified to bind with many different miRs, including, miR-7, miR-17, miR-214, miR-128, and miR-216b [2, 14, 55]. In one study, the expression level of *cir-ITCH* was determined in cancer tissues and paired adjacent noncancerous tissues of 78 lung cancer patients [56]. The results showed that the expression of *cir-ITCH* was significantly decreased in approximately 73% of the lung cancer tissues [56]. To investigate the interaction between *cir-ITCH* and the miRs, miR-7 and miR-214, the human lung cancer cell lines A549 and NCI-H460 were cotransfected with *cir-ITCH* plasmid and a constructed luciferase reporter of miR-7 or miR-214, and then the luciferase activity was detected. In both cell lines transfected with empty vector (control of *cir-ITCH* plasmid), luciferase activity was significantly decreased, in a concentration dependent manner, by a miR-7 or miR-214 mimic. However, no significance change in luciferase activity with the miR mimics occurred in cells with *cir-ITCH* hyper-expression. Thus, these results indicate that in lung cancer cells *cir-ITCH* can inhibit miR-7 and miR-214 through binding them as sponge [56].

In another study of non-small cell lung cancer (NSCLC) tissues from 101 patients, circRNA_100876 (circ-CER) was shown to be significantly upregulated compared to paired adjacent non-tumorous tissues [57]. Among the assessed clinicopathological parameters, there was a significant association between higher expression of circ-CER and both regional lymph node involvement and advanced tumor stage. Notably, the overall survival time of NSCLC patients with elevated circ-CER expression was significantly shorter than those patients with lower circ-CER expression [57]. Further research findings indicated that the circ-CER might be involved in tumor cell growth, progression and metastasis in NSCLC [58]. Therefore, circ-CER may prove to be a useful diagnostic marker and potential therapeutic target for the treatment of NSCLC in human patients [57].

### CircRNAs in hepatocellular carcinoma

There is increasing evidence linking circRNAs to the development of hepatocellular carcinoma (HCC). In an analysis of integrated high-throughput data, a number of circRNAs were shown to be linked to overexpression of miR-181a-3p, an inhibitor of the enzyme O(6)-Methylguanine-DNA methyltransferase (MGMT), which is known to be involved in DNA damage, thereby indicating a link between circRNAs and the progression of HCC through miR regulation [59]. In a more recent and extensive study, circRNA microarray detection was applied to both HCC tissues and adjacent normal tissues, identifying 61 differentially expressed circRNAs [60]. Of the three that were further confirmed by qRT-PCR, hsa_circ_0005075 demonstrated significant association with some clinicopathological factors of HCC patients [60]. Gene ontology and pathway analysis of hsa_circ_0005075 revealed that there was a strong relationship between this circRNA and cell adhesion, which is an important part of cancer cell proliferation and metastasis [60]. Moreover, larger sized HCC tumors showed greater levels of hsa_circ_0005075 expression, indicating that hsa_circ_0005075 may promote tumor growth. Therefore, hsa_circ_0005075 may have the potential to be a promising biomarker for HCC [60]. Based on the predictive program Arraystar (DNASTAR, Madison, WI, USA), which performs sequence variation analysis, four miRs, including miR-23b-5p, miR-93-3p, miR-581, and miR-23a-5p were identified as having binding sites within hsa_circ_0005075 [60]. Analysis of the hsa_circ_0005075 circRNA-miR-mRNA interaction network indicated that miR-23b-5p exhibited the largest interaction network [60]. Interestingly, miR-23b-5p has been shown to be downregulated in both gastric cancer and adenocarcinoma of the esophagus [61, 62]. It has been speculated that hsa_circ_0005075 acts as an miR sponge, suppressing the expression of miR-23b-5p in cancer [60]. While a high level of hsa_circ_0005075 expression in HCC tissues has been speculated to correlate with tumor progression, as mentioned above, the detailed molecular mechanisms by which hsa_circ_0005075 functions as a miR sponge to regulate the circRNA-miR-mRNA network in the process of HCC development needs further investigation.

Another circRNA, hsa_circ_0001649, has also shown decreased expression in HCC tissues compared with matched adjacent liver tissues [63]. Following the knockdown of hsa_circ_0001649 with siRNA, there was an increase in the expression level of the pro-metastatic matrix metalloproteinases (MPPs), MMP9, MMP10 and MMP13 [63]. This indicates that hsa_circ_0001649 is negatively correlated with the metastasis of HCC and may therefore also have the potential for use as a biomarker for diagnosing HCC [63].

Circular RNA profiling has also revealed that another circRNA, circHIPK3, is upregulated in liver cancer [20]. This circHIPK3, which is derived from Exon2 of the HIPK3 gene, has been reported in high abundance within both humans and mice [20]. This highly stable circRNA was shown to bind to multiple miRs, including a well-known tumor suppressor miR, miR-124, reducing its activity [20]. Therefore, targeting circHIPK3 may help to reduce patient HCC cell growth, like it has been shown in the huh7 hepatocarcinoma and HCT-116 colorectal carcinoma cell lines following circRNA silencing [20].

In a recent report examining tissue specific and developmental-stage-specific expression of human circRNAs, one of the examined circRNAs, CDR1as (also known as ciRS-7), a non-coding antisense transcript to the cerebellar degeneration-related protein 1 (CDR1), was identified to contain 63 conserved binding sites for miR-7 located within neural tissue, which acted to impair midbrain development in zebrafish due to inhibition of miR-7 function [2]. Interestingly, ciRS-7 has also been shown to be significantly upregulated in HCC tissues [64]. When ciRS-7 is knocked-down, there is a significant inhibition of HCC cell proliferation and invasion, which is likely due to the release of miR-7 [64, 65]. Thus, ciRS-7 was verified as a miR-7 sponge, acting to fine tune the expression of miR-7 targeted genes through an indirect regulation pathway.

### CircRNAs in gastric cancer

Using RNA-sequencing analysis, 180 circRNAs have been found to be differentially expressed in gastric cancer tissues compared with normal tissues [66]. Expression of the circRNAs, circPVT1, has been shown to be upregulated in gastric cancer [66]. A luciferase reporter assay has been used to confirm that circPVT1 acts as a sponge of the tumor suppressor miR-125 family, and also indirectly regulates the expression of the transcription factor, E2F2, which plays a crucial role in the control of the cell cycle and action of tumor suppressor proteins [66]. Consequently, the ectopic expression of circPVT1 may reduce the antineoplastic effects of miR-125b and E2F2. Therefore, circPVT1 has the potential to promote colony formation of gastric cancer cells *via* inhibition of miR-125 [66].

Another circRNA, hsa_circ_002059, has also been confirmed to be downregulated in 101 gastric cancer tissues that were compared with paired adjacent non-cancerous tissues [67]. The decreased expression of hsa_circ_002059 was notably related with distant metastasis, TNM stage, gender and age [67]. Together, these results suggest that hsa_circ_002059 may have the potential to be a biomarker for the diagnosis and staging of gastric cancer.

Hsa_circ_0000096, also known as circHIAT1, has also been found to be downregulated in gastric cancer tissues and gastric cancer cell lines compared with non-cancerous tissues and normal gastric epithelial cells [68]. Moreover, the siRNA knockdown of hsa_circ_0000096 has been shown to significantly inhibit cell proliferation and migration *in vitro* and *in vivo* [68]. The knockdown of hsa_circ_0000096 also blocked cell cycle progression and prevented the transition of cells from the G0/G1 to S phase within the gastric cancer cells [68]. Furthermore, knockdown of hsa_circ_0000096 in xenograft nude mouse models also suppress tumor growth. Generally, it is thought that within cancers oncogenes will be highly expressed, whereas tumor-suppressor genes are expressed at low levels. However, hsa_circ_0000096 appears to be an exception to this. This inconsistency may be explained by its interaction with miRs. Indeed, databases of circRNA, have indicated that hsa_circ_0000096 can interact with 17 different types of miR. Moreover, qRT-PCR results have confirmed that within a number of gastric cancer cell lines there was a decrease in miR-224, a CD40 regulator, and an increase in miR-200a, which targets E-cadherin, following the knockdown of hsa_circ_0000096 [68]. Together, the above data indicates that in addition to hsa_circ_002059, hsa_circ_0000096 may also be have a role in the future as a novel biomarker for the diagnosis of gastric cancer [68].

### circRNAs in colorectal cancer

As observed in lung cancer, the circRNA, *cir-ITCH*, has also been reported to be significantly down-regulated in 45 examined colorectal cancer (CRC) tissues [55]. In a study of the CRC cell lines, HCT116 and SW480, sponge activity of *cir-ITCH* was demonstrated towards miR-7, miR-20a, and miR-214 [55]. These miRs have been shown to downregulate a number of target genes that are mostly involved in regulation and execution of G1/S transition, including the pro-proliferative target gene cyclinD1 [55]. It has also been reported that the ectopic expression of *cir-ITCH* inhibits the expression of c-myc and cyclin D1, which are target genes of the Wnt/β-catenin signaling pathway [55]. This indicated that *cir-ITCH* may be involved in regulation of the Wnt/β-catenin signaling pathway, which is known to be important for the regulation of cell proliferation and migration [55]. In line with this, studies of *cir-ITCH* over-expression have demonstrated a decrease in cellular proliferation of both HCT116 and SW480 cells. Therefore, it is likely that *cir-ITCH* has an anti-proliferative role in HCCs [55].

Another circRNAs, hsa_circ_001569, has also been shown to highly expression in CRC when compared with non-cancerous tissue samples [69–71]. An assessment of two public databases (StarBase v2.0 and circBase) and three bioinformatic algorithms (TargetScan, Pictar and miRANDA) has shown that hsa_circ_001569 contains a binding site for miR-145, and that this miR has three target genes, E2F5, BAG4 and FMNL2, which it could inhibit through 3′UTRs promoter binding [1, 71, 72]. Previous studies have shown that E2F5 is a transcription factor that controls the gene expression of proteins involved in cell cycle control [73], BAG4 has been linked to cancer cell aggressiveness [74, 75], and FMNL2 promotes cell proliferation, motility, invasion, metastasis and epithelial-mesenchymal transition [76, 77]. By preventing the down-regulation of E2F5/BAG4/FMNL2, by miR-145, hsa_circ_001569 has been shown to promote CRC cell proliferation and invasion [71]. Subsequent studies have indicated that hsa_circ_001569 promotes proliferation by increasing the number of cells in the S and G2/M phases of the cell cycle [71]. Moreover, knockdown of hsa_circ_001569 in SW620 and LOVO cells has been shown to reverse invasive abilities [71]. However, unlike the other circRNAs that have been discussed, hsa_circ_001569 directly inhibits miR-145 transcriptional activity, rather than suppressing its expression [71].

Hsa_circ_0000069, which was identified by unsupervised hierarchical clustering analysis, also has been shown to be dysregulated in CRC [78], with over-expression identified in CRC tissues. Knockdown of hsa_circ_0000069 using siRNA, was reported to inhibit cell proliferation, migration, and invasion, as well as induce G0/G1 phase arrest of cell cycle in HT-29 cells [78].

Similarly, CRC tissues have also shown elevated levels of circ-BANP [79]. A knockdown of circ-BANP using siRNA was also shown to reduce proliferation and colony formation of the CRC cell lines, HCT116 and HT29. Moreover, p-Akt expression was reduced by this knockdown, indicating that circ-BANP-induced cell proliferation might involve the PI3K-Akt pathway, which is well known to be involved in cancer cell survival and cell cycle progression [79].

The circRNA hsa_circ_001988, has also been examined in 31 matched CRC tissues and normal colon mucosa [80]. Compared with the normal samples, the expression of hsa_circ_001988 was significantly downregulated in the CRC cell lines [80]. The results also demonstrated a significant correlation between the expression level of hsa_circ_001988 and cancer cell differentiation and perineural invasion. This is of particular importance because perineural invasion is a well-defined predictor of CRC patient outcomes and perineural invasion is known to be negatively associated with the survival time and recurrence of CRC in patients [19, 20]. These results indicate that hsa_circ_001988 may represent a potential novel biomarker of CRC prognosis [80].

Another recent study by Hsaio et al., used differential expression analysis of circRNAs in 48 colorectal tumor tissues compared to the corresponding adjacent non-tumor tissues [81]. The study identified circCCNB, circCDK13, and circCCDC66, which is formed from exons 8–10 of CCDC66, to be significantly elevated in the CRC tissues [81]. CircCCDC66 was selected by the authors for further investigation as its parental transcript possessed no known function. Interestingly, RT-PCR results from multiple cancer cell lines, including: colorectal (Caco-2, HCT116, HT-29, LS123), breast (MCF-7, MDA-MB-231, MDA-MB-468), pancreatic (BxPC-3, MIA PaCa-2) and cervical (HeLa) cancer cell lines, demonstrated circCCDC66 expression, which indicates that this circRNAs may be important for tumorigenesis [81]. Functional investigations both *in vivo* and *in vitro* revealed that circCCDC66 has an important role in a diverse number of pathological processes, including cell proliferation, migration, invasion, and anchorage-independent growth [81]. The study also identified 99 potential miRs which have binding sites within exons 8–10 of circCCDC66. This suggests that circCCDC66 may function as a miR sponge to protect a specific proto-oncogene, namely Myc, from miR activity in CRC. To confirm this, an *in vitro* transcription combined with *in vivo* circularization protocol was used to detect the sponge effects of circCCDC66. Three miRs (miR-33b, miR-93, and miR-185) were predicted to be sponged by circCCDC66. Further knockdown studies of circCCDC66 confirmed that it does function as miR sponge of miR-33b and miR-93 in CRC [81].

### circRNAs in esophageal cancer

Recent studies have uncovered several dysregulated circRNAs, including hsa_circ_001059, hsa_circ_000167, hsa_circ_0067934, and *cir-ITCH,* in esophageal squamous cell carcinoma (ESCC), which is the seventh most common cause of cancer related death in American Men [82–84]. In a study by Su et al., which identified over 3700 human circRNAs, two of these, hsa_circ_001059 and hsa_circ_000167, were observed at significantly different levels in the human radioresistant esophageal cancer cell line KYSE-150R, compared to parental cell line KYSE-150 [82]. Subsequent circRNAmiR coexpression network analysis showed that the above two circRNAs were the most important factors in the potential circRNA/miR network [82]. These results implicated the abnormally expressed circRNAs in the development of radiation resistance in the esophageal cancer cells [82].

Hsa_circ_0067934, is also markedly overexpressed in ESCC tissues and has been correlated with poor differentiation, as well the T stage (I-II), and TNM stage (I-II) [83]. Moreover, siRNA mediated silencing of hsa_circ_0067934 suppressed ESCC cell proliferation, migration and cell cycle progression [83]. Other clinical factors, such as lymph node metastasis or tumor size were not correlated with hsa_circ_0067934 expression. Considering that TNM staging is classily used to evaluate patient prognosis, whereby higher stages (stage III and IV) have a worse prognosis, it is possible that hsa_circ_0067934 may represent a potential marker of ESCC prognosis.

*Cir-ITCH,* which acts as tumor suppressor gene in lung cancer and CRC has also generally been shown to be down-regulated in ESCC, following the analysis of 684 ESCC and their adjacent noncancerous tissues [84]. The authors also identified that *cir-ITCH* acts as a sponge of miR-7, miR-17, and miR-214, thereby up-regulated the target gene ITCH, which negatively regulates the Wnt/β-catenin pathway *via* targeting dishevelled (Dvl) protein [85]. Importantly, it is known that the Wnt/β-catenin pathway has an important role in the carcinogenesis of many cancers, such as hepatobiliary cancer, lung cancer, breast cancer, and ESCC [86–89]. Collectively, this data exemplifies that role of *cir-ITCH* as a negative regulator of the Wnt/β-catenin signaling pathway, acting indirectly *via* sponging of miRs [84].

### circRNAs in breast cancer

Ductal carcinoma *in situ* (DCIS) accounts for approximately 20% of breast cancers detected by mammography [90]. While DCIS is generally considered to be highly curable, some women with DCIS will develop life-threatening invasive breast cancer, infiltrating ductal cancer (IDC), but the determinants of this progression remains largely unknown. This may be attributed to the same histological subtypes sharing similar gene expression patterns [91, 92].

An analysis of the expression of circRNAs in DCIS/IDC samples from five patients was assessed along with an invasive breast cancer cell line, MCF-7, using publicly available RNA-seq data and run on the bioinformatic detection pipeline, CIRCexplorer [92]. The results identified two circRNAs, hsa_circ_0122662 and hsa_circ_0001358, in the IDC samples and the MCF-7 cell line. Five possible miRs (miR-200c-3p, miR-200b-3p, miR-429, miR-376a-3p, and miR-376b-3p) were identified, using the Starbase human Pan cancer tool, that could interact with the hsa-circ-0001358 [92]. The first three, miR-200c-3p, miR-200b-3p, and miR-429, which belong to miR-200 family, could inhibit migration and invasion abilities of breast cancer cells [93, 94]. Following from this, genes known to participate in Epithelial Mesenchymal Transitions (EMT), which were known targets of the miR-200 family were examined, including ZEB1 (Zinc Finger E-Box Binding Homeobox 1), ZEB2 (Zinc Finger E-Box Binding Homeobox 2), VIM (Vimentin), BMI-1 (B lymphoma Mo-MLV insertion region 1 homolog), and FN1 (Fibronectin 1) [92]. However, no meaningful interactions were identified [92]. Interestingly, many circRNAs were found to be expressed in either DCIS or IDC, which could represent a measurable difference in dynamic expression. Therefore, differential circRNA expression may be worthy of further investigation, to help with the understanding of the molecular mechanisms of DCIS progression to IDC.

Circ-Seq analysis of circRNAs, has confirmed that circ-Foxo3 is also downregulated in breast cancer cell lines and breast cancer tissues when compared with noncancerous cell lines or normal tissues [21]. Overexpression of circ-Foxo3 in the breast cancer cell line, MDA-MB-231, significantly reduced proliferation and cell survival *in vitro* [95]. *In vivo* analysis of nude mice subcutaneously injected with MDA-MB-231 cells transfected with circ-Foxo3 demonstrated that circ-Foxo3 could inhibit tumor growth [95]. Moreover, TUNEL staining of tumor sections revealed that circ-Foxo3 transfected tumor cells demonstrate extensive cell death, suggesting expansive cell apoptosis in the tumors. In addition to this, 25 binding sites were detected in circ-Foxo3 for eight miRs, including miR-22, miR-136, miR-138, miR-149, miR-433, miR-762, miR-3614-5p, and miR-3622b-5p [21]. Transfection of these miRs into MDA-MB-231 cells decreased cellular apoptosis. Collectively these studies indicate that circ-Foxo3 exerts a fundamental biological role as an miR sponge.

### circRNAs in genitourinary system cancer

Bladder cancer is the most prevalent malignant tumor of the urinary system and is the 8th most common cause of cancer related death in men [96]. In a recent study by Zhong et al., high throughput microarray assays identified six circRNAs as differentially expressed in bladder cancer tissues when compared with paired non-cancerous tissues [97]. CircTCF25, circZFR, circPTK2, and circBC048201 were all significantly up-regulated, while circFAM169A and circTRIM24 were both down-regulated [97]. Bioinformatic analysis found MREs in circTCF25 that indicate binding sites for the miRs, miR-103a-3p and miR-107. DIANA-miRPath analysis revealed that both of these miRs are associated with the PI3K-Akt signaling pathway, which is frequently perturbed in cancer [97]. Further studies revealed that these miRs were also expressed at lower levels in the bladder carcinoma tissues [97]. Ectopic overexpression of circTCF25 was shown to promote proliferation and migration of the bladder cancer cells *via* inhibition of miR-103a-3p and miR-107 [97]. Previous studies have reported that miR-103a-3p and miR-107 can negatively regulate oncogenic factors, including CDK6 [98, 99]. Indeed, Western blot analysis of the miR sponge, circTCF25, demonstrated an increase in the protein level of CDK6. These results illustrate that the circTCF25-miR-103a-3p/miR-107-CDK6 network plays an important role in bladder cancer [97].

In a comprehensive analysis of differentially expressed profiles of circRNAs in four bladder carcinoma patients, 469 differentially expressed circRNAs were identified [100]. Gene Ontology analysis revealed that the gene expression profile of the linear counterparts of differentially over-expressed circRNAs favored protein modification processes, protein binding and cellular protein metabolic processes, while downregulated circRNAs favored molecular functions and catalytic activity [100]. 9 circRNAs where identified to have common binding sites for miRs, indicating the potential regulation of downstream target genes. These circRNAs included; circ-MYLK and circ-CTDP1, which have MREs for miR-29a-3p that targets DNMT3B, ITGB1, VEGFA and HAS3; and circ-PC, which has an MRE for miR-185-3p that targets ADD1and BAP1 [100]. As these target genes have been implicated in cancer cell development and progression, this circRNA-miR interaction offers a new perspective for the tumorigenesis mechanisms of bladder carcinomas.

In an assessment of clear cell renal cell carcinoma (ccRCC) tissues, expression of circRNA of Hippocampus Abundant Transcript 1(circHIAT1) was reported to be downregulated [101]. Moreover, circHIAT1 expression was found to be higher in non-metastatic ccRCC samples when compared to the matched metastatic ccRCC samples. Furthermore, ccRCC patients with higher circHIAT1 expression had a better overall survival rate than patients with lower circHIAT1 expression [101]. Analysis of circHIAT1 activity revealed direct binding to three miRs, miR-195-5p, MiR-29a-3p, and MiR-29c-3p. Interestingly, in contrast to the classical function of circRNAs serving as a “miR sponge”, circHIAT1 acted as an “miR reservoir”, increasing miR stability, and thereby resulting in reversal of androgen receptor-mediated ccRCC migration and invasion. In summary, the process of cell migration and invasion in ccRCC cells might be suppressed by the circHIAT1-mediated inhibition of miR-195-5p/29a-3p/29c-3p signaling pathways [101].

### circRNAs in other cancers

As we have seen, aberrant circRNAs have recently been implicated in a number of cancers, including lung, hepatic, gastric, colorectal, esophageal, breast and genitourinary cancers (Table 1). However, they are by no means limited to this set of cancers. In fact, this rapidly expanding field of research is bringing to light the important roles of circRNAs in many more cancer types.

**Table 1:** Identified circRNAs in a range cancers

Name	circRNA ID	Expression in cancer	Cancer	Sponge target	Function	regulated genes/pathways	Reference
CDR1as/ciRS-7	hsa_circ_0001946	upregulation	liver cancer	miR-7	oncogene	-	65
hsa_circ_0005075	hsa_circ_0005075	upregulation	liver cancer	miR-23b-5p, miR-93-3p, miR-581, miR-23a-5p	biomarker	-	60
circPVT1	hsa_circ_0001821	upregulation	gastric cancer	miR-125a/b	oncongene, biomarker	-	66
hsa_circ_001061	hsa_circ_0000069	upregulation	colorectal cancer	-	oncogene	-	78
hsa_circ_001569	hsa_circ_0000677	upregulation	colorectal cancer	miR-145	oncongene	-	71
hsa_circ_0067934	hsa_circ_0067934	upregulation	esophageal cancer	-	biomarker	-	83
hsa_circ_001059	hsa_circ_0000554	upregulation	esophageal cancer	miR-30c-1, miR-30c-2, miR-122, miR-139-3p, miR-339-5p, miR-1912	-	-	82
circTCF25	hsa_circ_0041103	upregulation	bladder cancer	miR-103a-3p, miR-107	oncogene, biomarker	PI3K-Akt signaling, CDK6, proliferation and migration	97
circHIPK3	hsa_circ_0000284	upregulation	liver cancer	miR-124, miR-152, miR-193a, miR-29a, miR-29b, miR-338, miR-379, miR-584 and miR-654	oncogene	-	20
circ-BANP	-	upregulation	colorectal cancer	-	oncogene	-	79
circRNA_100876/circ-CER	hsa_circ_0023404	upregulation	lung cancer	-	biomarker	-	57
circCCDC66	hsa_circ_0001313	upregulation	colorectal cancer, breast, pancreatic, cervical	miR-33b, miR-93	oncogene, biomarker	-	81
circRNA_100290	hsa_circ_0013339	upregulation	oral squamous cell carcinomas	miR-29	oncogene	G1/S growth cycle arrest, differentiation, apoptosis	108
cZNF292	hsa_circ_0004383	unknown	glioma	-	oncogene	Wnt/β-catenin signaling pathway, cell cycle progression	103
circ-Foxo3	hsa_circ_0006404	downregulation	breast cancer	miR-22, miR-136, miR-138, miR-149, miR-433, miR-762, miR-3614–5p, miR-3622b–5p	tumor suppressor	-	21, 95
CDR1as/ciRS-7	hsa_circ_0001946	downregulation	nervous system cancer, liver cancer	miR-7, miR-671-5p, miR-671-3p	biomarker	-	54, 64
hsa_circ_001599	hsa_circ_0001649	downregulation	liver cancer, gastric cancer	-	biomarker	-	63
hsa_circ_002059	hsa_circ_0000140	downregulation	gastric cancer	-	biomarker	-	67
hsa_circ_001988	hsa_circ_0001451	downregulation	colorectal cancer	-	biomarker	-	80
cir-ITCH	hsa_circ_0001141	downregulation	colorectal cancer, esophageal cancer, lung cancer	miR-7, miR-17, miR-20a, miR-214, miR-128, miR-216b	tumor suppressor	Wnt/β-catenin signaling pathway, cell cycle progression	55, 56, 84
hsa_circ_000167	hsa_circ_0000518	downregulation	esophageal cancer	miR-181a-2, miR-512-5p, miR-521, miR-556-5p, miR-663b, miR-1204	-	-	82
hsa_circ_0005785	hsa_circ_0005785	downregulation	pancreatic cancer	miR-181a/b	-	growth, migration and drug resistance	16
circHIAT1	hsa_circ_0000096	downregulation	gastric cancer, clear cell renal cell carcinoma	miR-195-5p/29a-3p/29c-3p, miR-224	biomarker, tumor suppressor	reversal of androgen receptor-mediated ccRCC migration and invasion	68, 101

Approximately 476 differentially expressed circRNAs have been revealed in 46 assessed gliomas tissues, as compared with control brain tissues [102]. Eight circRNAs, including circ_COL1A2, circ_PTN, circ_VCAN, circ_SMO, circ_PLOD2, circ_GLIS3, circ_EPHB4, and circ_CLIP2 showed significantly higher expression in glioblastoma (GBM) than in normal tissues [102]. It was speculated that these circRNAs will act as miR sponges to elevate the expression of genes involved in abnormal biological processes. However, this study requires future investigations to reveal the circRNA regulatory roles in GBM [102]. The circRNAs, cZNF292, has also been shown to participate in glioma cell growth and tube formation [103]. Silencing of cZNF292 expression was reported to inhibit glioma cell proliferation and halt cell cycle progression in U87MG and U251 cells *via* inactivation of the Wnt/β-catenin signaling pathway [103].

In laryngeal squamous cell cancers (LSCC), two circRNAs, hsa_circ_100855 and hsa_circ_104912, showed the greatest upregulation and downregulation, respectively, among the 698 identified dysregulated circRNAs [104]. The high level of hsa_circ_100855 was shown to correlate with the T3-4 stage, neck nodal metastasis and advanced clinical stage of LSCC, while the low level of hsa_circ_104912 related to the T3-4 stage, neck nodal metastasis, poor differentiation and advanced clinical stage. This report highlighted the potential for hsa_circ_100855 and hsa_circ_104912 as novel biomarkers for the diagnosis and progression of LSCCs [104].

In pancreatic ductal adenocarcinoma (PDAC), the expression profile of circRNAs between PDAC and the matched normal tissues identified 351 differentially expressed circRNAs by microarray analysis [16]. These included 209 up-regulated circRNAs and 142 down-regulated circRNAs. Among all the differentially expressed circRNAs, seven randomly selected circRNAs, including two up-regulated circRNAs (hsa_circ_0001946 and hsa_circ_0005397) and five down-regulated circRNAs (hsa_circ_0006913, hsa_circ_0000257, hsa_circ_0005785, hsa_circ_0041150, and hsa_circ_0008719), were examined by qRT-PCR to validate the microarray data. Moreover, circRNA-miR interaction analysis indicated that hsa_circ_0005785 has the potential capacity to bind to miR-181a and miR-181b [16]. Previous studies have demonstrated that miR-181a has a vital function in promoting pancreatic cancer growth and migration, while miR-181b has been associated with the resistance of pancreatic cancer cells to gemcitabine [105, 106]. This data supports a role for hsa_circ_0005785 in PDAC tumorigenesis and/or in mediating PDAC drug resistance.

An analysis of papillary thyroid cancers (PTC), using Arraystar Human circRNA Microarray to systematically profile and analyze the expression of circular RNAs, has revealed 88 significantly upregulated circRNAs and 10 downregulated circRNAs when compared to normal thyroid tissues [107]. Based on these dysregulated circRNAs and their predicted MREs, a network map of circRNA-miR interactions was constructed using Cytoscape. One of the downregulated circRNAs, hsa_circRNA_100395, showed interactive potential with two cancer-related miRs, miR-141-3p and miR-200a-3p [107]. This report suggests that the hsa_circRNA_100395-miR-141-3p/miR-200a-3p axis may be involved in the pathogenesis of PTC tumors [107]. However, this putative hypothesis is yet to be verified.

A cluster analysis of oral squamous cell carcinomas (OSCC), has identified 280 differentially expressed circRNAs, including 139 upregulated circRNAs and 141 downregulated circRNAs with a two-fold or greater change [108]. One of the circRNAs, circRNA_100290, was upregulated in OSCC by nearly seven-fold when assessed by microarray analysis, and four-fold when examined by qRT-PCR, when compared with non-cancerous matched tissue. Functional analysis found that a knockdown of circRNA_100290 could induce G1/S arrest in SCC9 cell lines, and this significantly inhibited cell proliferation. In accordance with this, si-circRNA_100290 decreased tumor growth *in vivo* nude mice models [108]. Cytoscape was used on the hsa_circRNA_100290-miR-target gene network to visualize their interactions based on the circRNA microarray data. The Arraystar predictive software listed miR-29b-3p, miR-29c-3p and miR-29a-3p as the top three most likely miR targets of circRNA_100290. The interaction between miR-29b and circRNA_100290 was then confirmed by a luciferase reporter assay. Together, the results indicated that circRNA_100290 may be a sponge of the miR-29 family, which are known to be involved in the regulation of cell proliferation, differentiation, and apoptosis [108].

In addition to the work on solid tumors, a role for circRNAs has also been shown in acute myeloid leukemia (AML). In a study of 115 human AML samples, hsa_circ_0004277 was reported to be downregulated [109]. Interestingly, a much lower expression level of hsa_circ_0004277 was found in newly diagnosed, untreated AML patients compared to the healthy controls, while patients that achieved complete remission following treatment had restored hsa_circ_0004277 expression, showing no difference compared to controls. Moreover, in the relapsed-refractory patients, downregulated hsa_circ_0004277 expression was again observed. Analysis of the molecular interaction between hsa_circ_0004277 and numerous miRs was also predicted, identifying miR-138-5p, miR-30c-1-3p hsa-miR-892b, hsa-miR-571, and hsa-miR-328-3p as potential candidates [109]. Downstream targets of these miRs, included SH3GL2, PPARGC1A, PIP4K2C, SH2B3, ZNF275, and ATP1B4 [109]. The mechanisms of which are yet to be established in AML. Therefore, it is possible that hsa_circ_0004277 might be a useful diagnostic biomarker in AML.

## CONCLUSIONS AND PERSPECTIVES

Since the first discovery of circRNAs, which were thought to be errors in RNA splicing, circRNAs are now regarded as ubiquitously expressed, abundant, and stable class of RNA molecules with a range of activities, including sponge, translation, biomarker, and regulation molecules. Recently, studies have highlighted the importance of circRNA dysregulation in a multitude of human cancers. As summarized in this review, circRNAs contribute to various biological processes within tumor cells, including proliferation, migration, invasion, and apoptosis. CircRNAs represent a novel class of diverse endogenous RNAs that regulate miR expression through harboring MREs. This circRNA-miR interaction can act by regulating downstream transcriptional activity of miR target genes. Interestingly, many of these miR target genes have been identified as oncogenes, indicating the importance of circRNA dysregulation in cancer. Previous studies have shown how miR expression can be regulated through mechanisms, such as DNA copy number variations, epigenetics, transcription factors and other mechanisms in cancer. Herein, we have reviewed a newly identified mechanism of miR regulation by circRNA occurring at the post-transcriptional level. Importantly, furthering our understanding of the regulatory mechanisms of circRNA on the expression of miRs will help provide new insights into cancer-cell development and may even prove to be clinically useful diagnostic and predictive biomarkers, as well as novel therapeutic targets for cancer patients.
